# Intracolonic Administration of Vancomycin in Intensive Care Unit Patients with Severe Clostridium Difficile Colitis

**DOI:** 10.7759/cureus.11573

**Published:** 2020-11-19

**Authors:** Alex Teixeira, Kartikeya Tripathi, Yesenia Greeff, Omar Sorour, Paul Mccallion, Garrison Davis, Khaled Sorour

**Affiliations:** 1 Gastroenterology and Hepatology, Signature Healthcare Brockton Hospital, Brockton, USA; 2 Gastroenterology, University of Massachusetts Medical School Baystate, Springfield, USA; 3 Gastroenterology and Hepatology, University of Massachusetts Medical School Baystate, Springfield, USA; 4 Internal Medicine, University of Massachusetts, Worcester, USA; 5 Anesthesiology, Signature Healthcare Brockton Hospital, Brockton, USA; 6 Internal Medicine, Signature Healthcare Brockton Hospital, Brockton, USA; 7 Critical Care Medicine, Signature Healthcare Brockton Hospital, Brockton, USA

**Keywords:** clostridium difficile infection, sepsis, colitis, vancomycin, colonoscopy, intracolonic vancomycin

## Abstract

Background: Clostridium difficile infection (CDI) is a major cause of antibiotic-associated diarrhea worldwide. The incidence of sepsis has been shown to be increasing due to severe or fulminant colitis. Oral vancomycin is the treatment of choice for CDI, but it is often ineffective in patients in the intensive care unit (ICU) due to poor intestinal motility. We present a review of eight cases with severe to fulminant CDI treated with adjunctive intracolonic vancomycin (ICV) administration.

Methods: A retrospective chart review identified patients in sepsis with severe colitis and positive Clostridium difficile toxin A or B. Patients who had failed standard therapy for CDI were given adjunctive ICV through an enteric tube, which was inserted via colonoscopy. To indicate the severity of patients, the patients selected had required vasopressor support.

Results: Eight patients (37.5% females) received this adjunctive treatment; the mean age was 73.25. The average Acute Physiology and Chronic Health Evaluation (APACHE) 2 score at the time of the procedure was 39. The median length of stay was 5.5 days, with in-hospital mortality of 37.5% and an average time to death of 1.33 days from the day of colonoscopy.

Conclusion: Colonoscopic decompression and administration of vancomycin for fulminant CDI using an enteric tube can have favorable outcomes in severely ill patients whose surgical options carry a high risk of mortality. Further larger randomized controlled trials are needed to evaluate its efficacy.

## Introduction

Clostridium difficile infection (CDI) is a major cause of antibiotic-associated diarrhea worldwide, and the incidence of fulminant colitis is on the rise [1,2]. CDI is responsible for approximately 30,000 deaths, leading to almost $5 billion in excess health care costs associated with acute care hospitalizations in the United States [3]. Of patients with CDI, 3%-8% develop fulminant colitis with a mortality rate of up to 57% [4,5]. Oral vancomycin therapy is the treatment of choice for Clostridium difficile colitis. However, it has shown to be ineffective in fulminant CDI, especially for patients in the intensive care unit (ICU), as poor intestinal motility prevents achieving adequate therapeutic concentrations of the antibiotic [6,7]. Concomitant use of rectal vancomycin with retention enemas fail to deliver proximally to the splenic flexure of the colon [8]. Effective treatment outcomes with intracolonic administration of vancomycin in critically ill patients with pseudomembranous colitis was first described as early as in 1993, but its use has been limited thus far due to risks of perforation of inflamed colon [9]. We present a review of eight cases where using intracolonic vancomycin (ICV) in severely ill patients in the ICU with CDI had preferable outcomes when compared to surgery.

## Materials and methods

We did a retrospective chart review from January 30, 2010 to December 31, 2012 for patients admitted in the ICU with CDI and septic shock. The study was approved by the Institutional Review Board. The diagnosis was based on the International Classification of Diseases, 9th revision (ICD-9) diagnosis codes from the electronic medical records at the Brockton hospital, Brockton, Massachusetts, USA. The case definition used was symptomatic diarrhea positive for Clostridium difficile toxins A and B in stool determined by enzyme immunoassay (EIA) or with endoscopic findings consistent with pseudomembranous colitis. The cases were de-identified using their account number and were given a unique alpha-numeric number. Data was captured on Microsoft Excel, which recorded patient demographics, clinical characteristics, hospital course, and treatment details.

The patients selected received adjunctive intracolonic administration with vancomycin. The severity of the disease was assessed clinically. Patients selected for the procedure were those who required high dose of vasopressors - i.e. over 20 mcg/min of norepinephrine and on vasopressin per protocol. All selected patients had failed standard therapy for CDI, including oral and rectal vancomycin, intravenous metronidazole and/or oral rifaximin. In addition, they received standard fluid and pressor management based on a standardized early goal-directed therapy-based protocol. A long enteric tube was inserted via colonoscopy and secured at the level of the cecum. Adjunctive ICV at a dose of 2 grams twice a day was delivered which was prepared in the hospital pharmacy.

In addition to standard demographic information, other forms of data collected included date of hospital admission, date of discharge, date of colonoscopy, length of hospital stay, quantification of diarrhea on admission, white blood cell (WBC) count on the day of colonoscopy, average WBC count, lactate on the day of colonoscopy, average lactate, Acute Physiology and Chronic Health Evaluation (APACHE) 2 score, colonoscopic findings and time to death. The primary outcomes were in-hospital mortality, resolution of shock, or resolution of leukocytosis. The secondary outcomes were the median length of stay, time to death, or discharge.

## Results

Eight patients received the adjunctive treatment and had either Clostridium difficile stool toxin positive or endoscopic findings of pseudomembranous colitis (Tables 1-2). The mean age of the included cases was 73.3 (SD ±11.9) with 37.5% females. The median length of stay in the hospital was 5.5 days with a range of 3 to 27 days. All eight patients had pseudomembranous colitis at the time of colonoscopy; one had necrosis. Average WBC count was 28.2 (±17.4) and an average lactate level of 3.6 (±2). The mean APACHE2 score at the time of procedure was 39, with in-hospital mortality among the patients who received adjunctive treatment of 37.5%. Their average time to death was 1.3 days from the day of the colonoscopy. None of the eight patients had known immediate complications from the procedure, including perforation. 

**Table 1:** Demographics, Clinical Characteristics of the Patients, Hospital Course and Treatment Details WBC: white blood cell; APACHE2: Acute Physiology and Chronic Health Evaluation 2 score.

Table 1. Demographics, Clinical Characteristics of the Patients, Hospital Course and Treatment Details								
Variable	Patient A1	Patient B1	Patient C1	Patient D1	Patient E1	Patient F1	Patient G1	Patient H1
Age	86	68	77	57	53	80	87	78
Gender	Male	Male	Female	Male	Female	Female	Male	Male
Date of admission	08/27/2012	05/26/12	01/25/12	05/09/11	05/07/11	04/26/11	10/06/10	09/19/10
Date of discharge*	09/05/12	06/01/12	02/01/12	05/10/11	05/13/11	05/06/11	10/09/10	10/26/10
Date of colonoscopy	09/05/12	05/29/12	01/26/12	05/10/11	05/09/11	04/27/11	10/07/10	10/13/10
Length of hospital stay	8	5	6	2	5	9	3	27
Diarrhea on admission	No	Yes	Yes	Yes	Yes	Yes	Yes	Yes
WBC count on day of colonoscopy	45.8	20.0	14.5	38.3	19.4	53.9	32.0	1.7
Lactate on the day of colonoscopy	2.0	1.7	4.2	>12.2	1.6	4.0	1.4	1.5
APACHE2 score	41	32	40	35	29	49	45	41
Colonoscopy findings	pseudomembrane	pseudomembrane	Ischemic colitis	pseudomembrane	pseudomembrane	pseudomembrane	pseudomembrane	Pseudomembrane necrosis
In hospital death	Yes	No	No	Yes	No	No	Yes	No
Date of death	09/05/12	-	-	05/10/11	-	-	10/09/10	-
Number of days- Admission to colonoscopy	8	2	2	2	1	2	2	18
Number of days- Colonoscopy to death	1	-	-	1	-	-	2	-

**Table 2:** Results WBC: white blood cell; APACHE2: Acute Physiology and Chronic Health Evaluation 2 score.

Variable	Result
Average Age (SD)	73.25 (11.91)
Males	62.5%
Females	37.5%
Median Length of stay (Range)	5.5 (3 to 27)
Average WBC count	28.2
Average lactate	3.6
Median lactate	1.9
Average APACHE2 score	39
In hospital mortality	37.5%
Average time to death after colonoscopy	1.33

## Discussion

CDI was first reported in 1978, and its incidence, recurrence, and virulence are on the rise [10]. It is established that CDI increases the cost of hospitalizations and raises the incidence of readmissions. It was reported in a study in 2016, that the total cost attributable to CDI management in the United States was nearly $6.3 billion. Treating CDI adds about $7,285 in hospital costs per patient, which does not include costs of readmissions [11]. The treatment and control of CDI has been a challenge and has not improved in the last several years. As per the Infectious Diseases Society of America (IDSA) and Society for Healthcare Epidemiology of America (SHEA) guidelines in 2018, high dose oral vancomycin in addition to retention rectal enema with or without intravenous metronidazole is recommended for fulminant CDI [12,13]. Despite these measures, it is frequently difficult to bring it to resolution, as ultimately it leads to colectomy and many times, death [14].

Due to lack of data from randomized controlled trials and the risks associated with the procedure, intracolonic use of vancomycin is not the standard of care in patients refractory to standard treatment. The only available literature is a retrospective case series, which makes it difficult to assess complications, recurrence, and success of treatment to formulate guidelines [15].

Interestingly, the mortality achieved by surgical intervention in these patients is comparable with this procedure. The mortality with surgery is similar or sometimes higher: on vasopressor support in sepsis with CDI, mortality ranges from 32%-57% after colectomy [16,17]. We propose that adjunctive use of ICV could in certain cases obviate the need for surgery and salvage the colon, especially elderly patients with multiple co-morbidities who are not surgical candidates. Of the five patients who were older than 70 years of age, the presence of hemodynamic instability and leukocytosis or leukopenia predict a mortality rate of 57.1% with surgery, whereas the mortality rate in our series for those patients was 40% [17]. 

Unfortunately, the latest guidelines by IDSA and SHEA do not mention the use of adjunctive ICV, presumably due to limited scholarly work and retrospective data available [6,12,18]. As per these guidelines, segmental or subtotal colectomy or loop ileostomy with antegrade vancomycin lavage is usually the next course in the management. However, the earlier guidelines noted its use for fulminant CDI, but did not necessitate or elaborate a clinical criterion for patient selection. It is important to ascertain that most of these patients, especially in the ICU, have other co-morbidities limiting their surgical options and exponentially increase their risk of postoperative morbidity and mortality. It would be reasonable to mention that there are not any surgical guidelines for patient selection, and more often than not, the decision is made clinically. Adjunctive use of ICV could be an alternative in these patients to avoid surgery or be attempted when surgery is not feasible given other risks [19].

There are several strengths to this review. We emphasize the use of ICV could be an alternative to surgery in patients, especially in the ICU. It is evident from the review that early instillation of ICV could decrease mortality. It could be done as early as on day two. Mortality was directly proportional to the delay in the procedure. This procedure was done in a community hospital without any reported complication of the procedure itself, including perforation.

This review has some limitations. As with all retrospective observational study, data was retrieved from the existing records, which could have missing information. However, given the design of the study and data collection, the likelihood of missing data was minimal. Follow-up records were not obtained when the patient was discharged from the hospital. It would be useful to follow these patients and document resolution or assess for recurrence. Therefore, the disease severity and intervention with ICV of these patients may have been skewed. We did not have a control group, as these patients were too sick for a placebo-controlled trial, and it would be unethical not to intervene. Use of ICV can probably be compared to patients with APACHE2 scores of 39, however, whose expected mortality is 82% percent. Finally, the small number of patients in the review and a single-center data point to the need for large randomized control trials to compare the efficacy of ICV to surgery or conservative options.

## Conclusions

The incidence of CDI has been increasing and associated readmissions, in turn, increase healthcare expenses. Severe sepsis with CDI, leads to inability to perform further intervention since these patients are not surgical candidates for colectomy. Ultimately, the mortality of these patients is increased in the absence of intervention. When other modalities fail, surgery is not an option and goals of care are discussed, we suggest that colonoscopic assisted colonic/enteric tube administration of vancomycin could be used in these patients.
